# Mastering your fellowship: Part 2, 2022

**DOI:** 10.4102/safp.v64i1.5504

**Published:** 2022-04-13

**Authors:** Mergan Naidoo, Klaus B. von Pressentin, Tasleem Ras, Ts’epo Motsohi

**Affiliations:** 1 Department of Family Medicine, College of Health Sciences, University of KwaZulu-Natal, Durban, South Africa; 2 Division of Family Medicine, Faculty of Medicine, University of Cape Town, Cape Town, South Africa; 3 Department of Family and Emergency Medicine, Faculty of Health Sciences, Stellenbosch University, Cape Town, South Africa

**Keywords:** family physicians, FCFP (SA) examination, family medicine registrars, postgraduate training, national exit examination

## Abstract

The series, ‘Mastering your Fellowship’, provides examples of the question format encountered in the written and clinical examinations, Part A of the Fellow of the College of Family Physicians South Africa (FCFP SA) examination. The series is aimed at helping family medicine registrars prepare for this examination. Model answers are available online.

## Introduction

This section in the *South African Family Practice* journal is aimed at helping registrars prepare for the Fellow of the College of Family Physicians South Africa (FCFP SA) Final Part A examination and will provide examples of the question formats encountered in the written examination. These question formats include multiple-choice question (MCQ) in the form of single best answer (SBA – Type A) and/or extended matching question (EMQ – Type R), short answer question (SAQ), questions based on the critical reading of a journal (evidence-based medicine) and an example of an objectively structured clinical examination (OSCE) question. Each of these question types is presented based on the College of Family Physicians blueprint and the key learning outcomes of the FCFP programme. The MCQs will be based on the 10 clinical domains of family medicine, the SAQs will be aligned with the five national unit standards and the critical reading section will include evidence-based medicine and primary care research methods.

This edition is based on unit standard one (critically reviewing new evidence and applying the evidence in practice, principles of self-care and leading a clinical governance team), unit standard two (evaluate and manage a patient according to the biopsychosocial approach) and unit standard four (facilitate the learning of others). The domain covered in this edition is Trauma. We suggest that you attempt answering the questions (by yourself or with peers or supervisors), before finding the model answers online: http://www.safpj.co.za/.

Please visit the Colleges of Medicine website for guidelines on the Fellowship examination: https://www.cmsa.co.za/view_exam.aspx?QualificationID=9.

We are keen to hear about how this series is assisting registrars and their supervisors in preparing for the FCFP (SA) examination. Please email us (naidoom@ukzn.ac.za) your feedback and suggestions.

## Multiple-choice question: Single best answer

A 23-year-old male motorcyclist is involved in a motor vehicle accident and is brought into your rural district hospital’s emergency centre (EC). He complains of abdominal pain and pain in his limbs. The patient is anxious but alert. Both his lower limbs look deformed. His vital signs are as follows: Blood Pressure (BP) = 90/60 mmHg, pulse rate (PR) = 125/min, respiratory rate (RR) = 24/min, oxygen saturation (SpO2) = 96% in room air. He has generalised abdominal tenderness with guarding. You immediately set up two wide bore intravenous lines and administer 1 litre of Ringers Lactate. What would you administer as the next most appropriate step?

- Freeze-dried plasma
- Packed cells
- Ringers Lactate
- Voluven
- Whole blood

*Answer b)*

**Discussion:** The Advanced Trauma Life Support (ATLS) algorithms for managing shock changed in the 2018 edition after new data emerged that using more than 1.5 litres of isotonic resuscitation fluid is associated with increased mortality. Firstly, on presentation, it is important to recognise that this patient has Class III haemorrhagic shock from the clinical features present (See Table 1).

**TABLE 1:** Classification of haemorrhagic shock.

Parameter	Class I	Class II	Class III	Class IV
Blood loss (% blood volume)	15	15–30	31–40	> 40
Pulse rate	< 100	100–120	120–140	> 140
Systolic blood pressure	Normal	Normal	Decreased	Decreased
Pulse pressure	Normal or increased	Decreased	Decreased	Decreased
Respiratory rate	14–20	20–30	30–40	> 35
Urine output (ml/hr)	> 30	20–30	5–15	Negligible
Mental state	Slightly anxious	Mildly anxious	Anxious, confused	Confused, lethargic
Base deficit	0 mEq/L to – 2 mEq/L	−2 mEq/L to – 6 mEq/L	−6 mEq/L to – 10 mEq/L	−10 mEq/L or less
Need for blood products	Monitor	Possible	Yes	Massive Transfusion Protocol

*Source:* Mutschler M, Nienaber U, Brockamp T, et al. A critical reappraisal of the ATLS classification of hypovolaemic shock: Does it really reflect clinical reality? Resuscitation. 2013;84(3):309–313. https://doi.org/10.1016/j.resuscitation.2012.07.012

Class III shock implies that 31% – 40% of the blood volume was lost and this could have been due to intra-abdominal trauma, pelvic fractures and lower limb fractures. This translates into approximately 2 litres of lost blood volume. The ATLS principles require that early control of haemorrhage is vital to the management of the severely injured patient. Splinting of the limbs and the judicious use of tourniquets placed above the area of injury in uncontrolled haemorrhage are options that one can consider. The early resuscitation with blood is recommended in patients with evidence of Class III and IV haemorrhage and should begin after one litre of warmed crystalloid fluid. Severe shock from trauma can predispose to coagulopathy, dilution from large volume crystalloid infusion and hypothermia. There is evidence that intravenous tranexamic acid one gram given in the pre-hospital setting and repeated in the EC may confer some benefits to the resuscitative efforts. In addition, to aid respiratory efforts and improve the delivery of oxygen to the patient, oxygen should be administered to this patient.

A complete primary survey and resuscitation should be accompanied by a thorough secondary survey and this must include an assessment of head, neck, spine, thorax, abdomen and musculoskeletal system. This should be accompanied by appropriate investigations (both blood and radiological investigations). Local district hospitals should also seek to improve their diagnostic acumen by acquiring ultrasound machines for the EC. The Extended Focused Assessment with Sonography in Trauma (E-FAST) is a useful clinical skill and included in the FCFP portfolio of learning. This will help identify the source of intra-abdominal trauma and determine the need for urgent surgical interventions.

Ideally in haemorrhagic shock one would seek to provide equal quantities of plasma, red blood cells and platelets. All IV fluids should preferably be warmed prior to administration. However, context matters and in most district hospitals ready access to whole blood is not feasible, but emergency O negative or positive blood is available, hence the choice of answer.

It is important to note that adequate stabilisation may not occur due to ongoing blood losses, so early consultation with the regional or tertiary referral centre is needed for management advice. Also important is the ongoing monitoring and response to abnormal clinical findings and packaging of the patient for transfer.

### Further reading

American College of Surgeons. Advanced Trauma Life Support student course manual. 10th ed. Chicago, IL: American College of Surgeons; 2018.

## Short answer question: The family physician’s role as a teacher

You are the family physician in charge of a small district hospital. During your weekly midday casualty teaching round, the community-service medical officer (CSMO) presents a patient who is lying awake in a trauma bed. He was a passenger sitting on the window side of a minibus taxi which was hit from the side by a car at an intersection. One of the passengers in the car had died and five passengers in the minibus taxi had sustained fractures of arms or limbs. The CSMO states that the passenger sustained an undisplaced midshaft fracture of the right tibia which was confirmed on X-ray and had numerous closed soft tissue injuries of the right chest and arm. He also sustained lacerations on the right parietal scalp and left lower leg. The patient had consumed six beers before boarding the taxi and has no past medical or surgical history. All his current vitals are normal. The CSMO reports that a below-knee circular plaster of paris (POP) was placed and that the patient had received paracetamol and ibuprofen for pain. The lacerations were sutured with 3/0 Nylon. He indicates that the plan was to fit the patient for crutches, then discharge the patient on analgesia for follow up in 1 week to remove sutures and check the POP. He plans to have the POP removed in 8 weeks.

1. List four additions and modifications you would make to this management plan. (4 marks)
2. List three additions and modifications you would make to his discharge plan. (3 marks)
3. Identify three broad learning needs in the management of this case. (3 marks)
4. Outline an approach/tool you use to structure this teaching round to facilitate learning for the community service officer. (5 marks)
5. Outline the educational (not operational) steps you would take to design a continuous professional development (CPD) teaching session on fracture management to the community service doctors in your hospital (5 marks)

Model Answers

### 1. List four additions and modifications you would make to his immediate management plan. (4 marks)

The current management must also include (any four):

- Spinal motion restriction with rigid neck brace/blocks/blanket and spinal board until the cervical spine (CS) is cleared.
- Re-check and document neurology and vascular checks before and after CS immobilisation.
- Request a thorough CS examination and X-rays because the patient is likely to be intoxicated.
- Clear chest, abdomen, and pelvis for internal injuries on examination including a chest X-ray.
- Extend the POP to above knee and include ankle and foot to the level of metatarso-phalyngeal (MTP) joint. Reassess swelling and consider a split cast/backslab.
- Consider augmenting pain management with morphine while still in hospital and during POP replacement.
- Request tetanus toxoid to be given if indicated.
- Assess patients’ level of intoxication and consider keeping patients in the hospital longer for neurological observation.

### 2. List three additions and modifications you would make to his discharge and follow-up plan. (3 marks)

Any three:

- Once ready for discharge, request a review in 1 day for a circulation check of the leg
- Give patient written information in their language on POP danger signs and symptoms
- Request a subsequent review of the POP for position and tightness in 2 weeks with a repeat X-ray
- Removal of POP in 12 weeks with referral to physiotherapy for rehabilitation
- Remove sutures on lower leg in 12 days to 14 days and not 1 week
- Ensure that the plan is written for the patient and includes how to care for the POP and crutches.
- Make a note to enquire about alcohol use at follow up visit
- Enquire about and issue a medical certificate

### 3. Identify three broad learning needs in the management of this case. (3 marks)

- Primary Survey in Trauma (includes c-spine assessment and clearance)
- Principles of fracture management and care

Plus any one of the following:

- Principles of sutured wound care
- Principles of pain management in trauma
- Principles of managing intoxicated trauma patients

### 4. Outline an approach you would use to structure this teaching round to facilitate learning for the community service officer. (5 marks)

- Pendleton’s feedback model:
  ■ Ask them what they thought they did well in the patient’s management, ensuring they focus on strengths
  ■ Discuss what went well and reinforce it with your own observations of strengths
  ■ Ask them what they could do even better
  ■ Discuss what they have identified and add your own additional observations.
  ■ Outline a plan on how they can improve in future

OR

- 1-min preceptor:
  ■ Confirm commitment to an assessment.
  ■ Probe for further supporting evidence of the assessment
  ■ Teach general rules of clinical reasoning/management focusing on one or two important gaps
  ■ Reinforce what was done correctly
  ■ Correct mistakes

### 5. Outline the educational (not operational) steps you would take to design a continuous professional development (CPD) teaching session on Fracture management to the community service doctors in your hospital. (5 marks)

- Consult the doctors on their *learning needs* for the topic (1 mark)
- Identify achievable *learning outcomes* for the needs and cover *knowledge, skills, and attitude* (1 mark)
- Create a detailed *outline of the content*/*information* that will be covered (1 mark)
- Select the *appropriate teaching methods* for the learning outcomes and content (1 mark)
- Create a *qualitative and quantitative feedback/evaluation* form for the participants (1 mark)

### Further reading

- American College of Surgeons. Advanced Trauma Life Support student course manual. 10th edition. Chicago, IL: American College of Surgeons; 2018.
- Kapp P. Chapter 111: How to apply plaster casts and splints. In: Mash B, Blitz J, editors. The South African family practice manual. 3rd ed. Pretoria: Van Schaik Publishers, 2015; p. 360–362.
- De Villiers M. Chapter 174: How to plan and implement a teaching activity or continuing professional development meeting. In: Mash B. Blitz J, editors. The South African family practice manual. 3rd ed. Pretoria: Van Schaik Publishers, 2015; p. 594–596.
- Blitz J. Chapter 9: Developing the primary care team. In: Mash B, editor. The handbook of family medicine. 4th ed. Cape Town: Oxford University Press, 2017; p. 386–405.

## Critical appraisal of research

Read the accompanying article carefully and then answer the following questions (*total 30 marks*). As far as possible use your own words. Be guided by the allocation of marks concerning the length of your responses.

- Whitaker J, Nepogodiev D, Leather A, Davies J. Assessing barriers to quality trauma care in low and middle-income countries: A Delphi study. Injury. 2020;51(2):278–285. https://doi.org/10.1016/j.injury.2019.12.035

1. What research question did the authors attempt to answer in this study? Comment on whether this was a clearly focused question concerning the PICO framework (Population of interest, Intervention or Issue of Interest, Comparison intervention of interest, primary Outcome of interest). (6 marks)
2. In your own words, describe your understanding of what the Delphi technique aims to achieve and what role it plays in health sciences research. (3 marks)
3. Comment critically on the expert panel used in this Delphi study in terms of the number of experts, selection of experts and composition. (6 marks)
4. How was consensus defined, measured and reached in this Delphi study? (5 marks)
5. Use the acronym READER (Relevance, Education, Applicability, Discrimination, Evaluation and Reaction) to analyse this article’s applicability to your own context (take-home message). (10 marks)

Total: 30 marks

Model answers

### 1. What research question did the authors attempt to answer in this study? Comment on whether this was a clearly focused question concerning the PICO framework (Population of interest, Intervention or Issue of Interest, Comparison intervention of interest, primary Outcome of interest). (6 marks)

The study aimed to develop expert consensus on the most important barriers (within a Three Delays framework) to accessing injury care in low- and middle-income countries (LMICs), which are important to assess and to effectively evaluate an LMIC trauma care health system. The Three Delays framework has been widely adopted in maternal, neonatal and child health; the authors state that this framework has been proposed for evaluating emergency healthcare systems more generally including trauma. The framework considers barriers resulting in delays seeking care (Delay 1), reaching care (Delay 2) and receiving appropriate care (Delay 3).

- Using the PICO framework for this study (*see additional information below*):
  ■ The Patient problem (*P*) relates to patients in need of injury or trauma care.
  ■ The issue of interest (I): the most important barriers to accessing this care.
  ■ Although there is no explicit comparison intervention of interest, the context (*C*) is that of emergency healthcare systems in LMICs.
  ■ The outcome of interest (O) would be the grading (assessment of importance according to the Three Delays framework) via expert consensus, on which of these barriers would be deemed to be most important to assess and to inform health system assessment development.
- This study, therefore, aimed to answer a question well aligned with the Delphi technique. The study aimed to steer a group of experts towards consensus regarding a wide-ranging question in the LMIC context.

*Additional information* (*not part of the model answer*): The PICO framework (Patient group, Patient Problem or Population of interest, Intervention or Issue of Interest, Comparison intervention of interest, primary Outcome of interest) is generally used to help frame or focus the research question. The framework may be tailored to the research question type (treatment, prevention, diagnosis, prognosis, or aetiology) or study design (quantitative compared to qualitative).

### 2. In your own words, describe your understanding of what the Delphi technique aims to achieve and what role it plays in health sciences research. (3 marks)

- The Delphi technique is a method for structuring a group communication process that aims to develop expert-based judgment around a certain question or topic, grounded on the principle that it would be possible and of value to reach consensus.
- It is an acceptable method to obtain a consensus among a group of independent experts, using a distinct set of group interactions, including ensuring anonymous interactions between experts, employing multiple rounds of questioning and the provision of feedback to the group between rounds.
- In health sciences research, it is often used in the following situations to determine, forecast, and discover group attitudes, needs, and priorities:
  ■ To develop clinical practice or reporting guidelines.
  ■ To explore and achieve consensus on disputed topics or understanding areas of limited research.

### 3. Comment critically on the expert panel used in this Delphi study in terms of the number of experts, selection of experts and composition. (6 marks)

The selection of experts as panellists represents a key step in the Delphi method. These panellists represent a group of participants who were selected for their expertise on the research topic. The following three key aspects should be considered when appraising the expert panel composition:

- *Number of experts:* It is important to record the number of experts invited. It is also important to describe how the number of experts changed over the different rounds.
  ■ In the results section, the authors confirmed that 49 participants expressed interest in the study and were invited into round 1. Figure 2, in Whitaker et al. 2020, describes the flow chart of progress of participants through the study. There were 37 eligible responses to round 1, 30 to round 2 and 27 to round 3 (see Figure 2 in Whitaker et al. 2020).
- Selection of experts: Which categories of experts were included and how were the experts identified and approached? It is also important to note how the experts were chosen (e.g. willingness to participate, expertise, or membership in an organisation).
  ■ In Whitaker et al. 2020, the authors described the identification of experts in the methods section, as ‘individuals with a holistic health systems overview of barriers to accessing trauma care were invited to contribute’ (no specific discipline mentioned).
  ■ In terms of selecting the experts for this Delphi study, the authors identified potential participants through international injury care and health system research organisations including Health Systems Global network, the Primary Trauma Care network, the College of Surgeons of East, Central and Southern Africa (COSECSA), the GlobalSurg Collaborative and other personal contacts. Potential participants were informed of the study through both direct face to face and email communication and via electronic advertisement.
  ■ Eight round 1 participants had responded to direct invitation, while 29 participants had responded to a request through a professional network.
- *Expert panel composition:* One should evaluate the data provided in the paper which describes the invited experts (i.e. speciality, age and years of experience), the composition of the panel (e.g. patients, healthcare professionals, managers, academics), and whether the panel included professionals from single or multiple specialities.
  ■ In terms of evaluating the panel composition, the researchers required experts to have at least 6 months of clinical experience treating injured patients in LMICs in the preceding 2 years or two or more publications on LMIC health systems research or trauma research in the preceding 2 years. It would be useful to understand the authors’ decision to use a 2-year and/or two or more publications cut-off to determine sufficient expertise to help provide consensus on the research question. The rationale for these cut-scores was not explained in the paper. It is good, however, that the authors valued a combination of clinical and research expertise in the subject matter.
  ■ Experts from geographically and economically diverse settings were approached to ensure generalisability across LMIC contexts. In the results section, Table 2 provides an overview of the panellists in terms of their work setting (rural vs urban), gender, area of expertise, and country of work (World Bank Income Classification). The data in Table 2 shows that the panellist composition was skewed towards an urban work setting, male gender and middle income (both lower and upper) country settings.
  ■ As mentioned above, no specific disciplines or professions were mentioned. This limits our ability to understand how heterogenous the panel composition is in terms of disciplinary/profession representation (healthcare professions, scientists and managers). However, there seemed to be a good balance between clinical and research expertise in the panel.

To conclude the critical evaluation of the method in which the expert panellists were chosen, it seems to be a combination of willingness to participate and having membership in an organisation or network. The experts were approached deliberately by the researchers (a non-randomised selection process). A grading system was used to determine expertise (clinical and/or research) as well as country representativeness in terms of World Bank classification. The authors noted in the results section, that the panel composition between the rounds and the balance between work settings, gender and area of expertise remained similar, although all participants from low- and high-income countries completed all three rounds. As noted above, the panel composition was skewed in terms of certain key criteria and this might influence the usefulness of the study findings.

### 4. How was consensus defined, measured and reached in this Delphi study? (5 marks)

**FIGURE 1:**
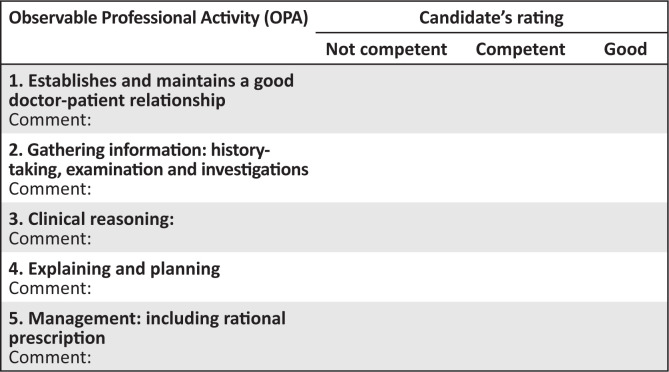
Marking template for consultation station.

- The definition of consensus should be defined *a priori* (during the design of the study).
- The authors designed a three-round modified Delphi study.
  ■ Consensus was measured and reached mainly during round 2 and 3, as round 1 consisted of a more open-ended questionnaire used by the expert panel to generate a list of proposed barriers (this list was synthesised by the researchers into 20 distinct conceptual barriers potentially delaying care following injury in LMICs and categorised within the Three Delays framework).
  ■ During round 2, a 5-point Likert scale (strongly agree, agree, neither agree nor disagree, disagree or strongly disagree) was used to assess the agreement on as to whether each of these barriers was easy to assess (feasible), likely to delay care for a significant proportion of injured persons (large scale), likely to cause avoidable death or disability for affected injured persons (high impact), or readily changed to improve care for injured patients (modifiable).
  ■ For round 3, the a priori primary outcome was the proportion of participants strongly agreeing or agreeing with each barrier’s four components (feasible, large scale, high impact, modifiable).
- Consensus agreement was defined as ≥ 70% participants strongly agreeing or agreeing, while consensus disagreement was defined as ≥ 70% participants strongly disagreeing or disagreeing. The number of components achieving consensus for each barrier was calculated. As a secondary outcome, each of the 5 points on the Likert scale was also assigned a score from 2 to –2 to allow further comparison and differentiation between each barrier. Figure 1 in the paper illustrates the process of calculating the primary and secondary outcomes.
- Overall, 7 of 20 barriers achieved consensus (> 70%) agreement across all 4 components in round 2. Consensus agreement had been reached for all 4 components in 11 of 20 barriers in round 3. However, following the completion of round 3, no consensus of disagreement was achieved for any barrier component. Table 4 in the paper presents the results in order of round 3 average barrier scores.

### 5. Use the acronym READER (Relevance, Education, Applicability, Discrimination, Evaluation and Reaction) to analyse this article’s applicability to your own context (take-home message). (10 marks)

The READER format may be used to answer this question:

- Relevance to family medicine and primary care?
- Education – does it challenge existing knowledge or thinking?
- Applicability – are the results applicable to my practice?
- Discrimination – is the study scientifically valid enough?
- Evaluation – given the above, how would I score or evaluate the usefulness of this study to my practice?
- Reaction – what will I do with the study findings?

The answer may be a subjective response but should be one that demonstrates a reflection on the possible changes within the student’s practice in the South African public healthcare system. It is acceptable for the student to suggest how their practice might change, within other scenarios after graduation (e.g. general private practice). The reflection on whether all important outcomes were considered is, therefore, dependent on the reader’s own perspective (is there other information you would have liked to see?).

A model answer could be written from the perspective of the family physician employed in the district health system:

- This study is *relevant* to the African primary care context, as trauma represents a major public health problem, especially in LMICs. The authors stated that good trauma care reflects wider emergency health system performance and cite the Lancet Global Health Commission on High Quality Health Systems, which described the disparity between the global injury burden and the limited available data on care quality provided by health systems. As such, better assessment of trauma care systems in Africa should be a research and health service priority.
- The *educational* value is moderate to good, as many of the conceptual barriers to injury care are well known. However, the Three Delays framework and its repurposing to classify barriers to injury care is quite novel.
- The study should be *applicable* to the family physician employed in the district health system, as they will be tasked with providing leadership in the local health services team to strengthen and coordinate acute injury care. This necessitates a community-oriented approach, as appreciating and addressing barriers to trauma care will enable intersectoral collaboration with community partners to prevent avoidable death or disability. The Three Delays framework and the grading of barriers will help the family physician to engage with the health services clinical management team to prioritise interventions. This framework may also be used in morbidity and mortality reviews and planning quality improvement interventions.
- In terms of *discrimination*, the methods to reach consensus among the group of experts are well described. The grading criteria to describe each of the barrier’s four components (feasible, large scale, high impact and modifiable) were applied rigorously based on an *a priori* primary and secondary outcomes. The expert panel composition review raised a few concerns, however, as its composition appeared to be skewed towards male experts from urban, middle income (both lower and upper) country settings. However, there seemed to be a good balance between clinical and research expertise in the panel. The disciplinary heterogeneity (number of stakeholder types) of the panel members was not described sufficiently.
- In terms of *evaluating* the credibility and acceptance of the Delphi study findings, the panel composition should reflect the full range of stakeholders who have an interest in the results of the study. Moreover, different stakeholders often have very different points of view, which enrich the results of the Delphi procedure. For a South African public sector family physician, the study composition may be sufficient in terms of LMIC representation (especially middle-income country classification). The methods to obtain consensus in rounds 2 and 3 are well described and appear to have been applied in a rigorous manner. The authors argued that the panel size and the response drop-out rate were acceptable (although from the four South African panellists only two participated in round 2 and none participated in round 3). In the limitations section, the authors mention the slight dominance of participant African experience (9 of 21), which may have resulted in bias towards issues pertinent to that continent where, for example, staff workforce density is particularly low. Furthermore, the authors argue that the observed gender imbalance may partly reflect the male dominance in specialities such as surgery seen in high- and low-income settings. Other limitations voiced by the authors were lack of input from participants from more diverse clinical settings (such as non-trauma emergency care and primary care), English limiting participation and a lack of a patient voice regarding barriers to care.
- In terms of *reaction,* the study may be discussed with the local clinical management team and used as the basis for creating awareness regarding the barriers to injury care, which may lead to health service interventions and/or applied research activities. The expert-guided prioritisation of the Delphi process may be especially relevant for focused interventions to address the barriers which were considered feasible to assess, impacting many people, causing avoidable mortality and morbidity, and are potentially amenable to change.

### Further reading

- Niederberger M, Spranger J. Delphi technique in health sciences: A map. Front Public Health. 2020;8:457. https://doi.org/10.3389/fpubh.2020.00457
- Jünger S, Payne SA, Brine J, Radbruch L, Brearley SG. Guidance on Conducting and REporting DElphi Studies (CREDES) in palliative care: Recommendations based on a methodological systematic review. Palliat Med. 2017;31(8):684–706. https://doi.org/10.1177/0269216317690685
- McMillan SS, King M, Tully MP. How to use the nominal group and Delphi techniques. Int J Clin Pharm. 2016;38(3): 655–662. https://doi.org/10.1007/s11096-016-0257-x
- Iqbal S, Pipon-Young L. Methods: The Delphi method. Psychologist. 2009;22(7):598.
- Boulkedid R, Abdoul H, Loustau M, Sibony O, Alberti C. Using and reporting the Delphi method for selecting healthcare quality indicators: A systematic review. PLoS One. 2011;6(6):e20476. https://doi.org/10.1371/journal.pone.0020476

## Objectively structured clinical examination scenario

### Objective of the station

This station tests the candidate’s ability to care for a patient with concussion.

### Type of station

Integrated consultation.

### Role player

Young man.

### Instruction to the candidate

- You are the family physician working in the EC. The following patient is brought by his coach, having just sustained a head injury during a rugby match.
- Please consult with the patient and manage accordingly.
- Relevant clinical examination findings will be provided on request.

### Instructions for the examiner

- This is an integrated consultation station in which the candidate has 15 min.
- Familiarise yourself with the assessor guidelines that details the required responses expected from the candidate.
- No marks are allocated. In the mark sheet, tick off one of the three responses for each of the competencies listed. Make sure you are clear on what the criteria are for judging a candidate’s competence in each area.
- Please switch off your cell phone.
- Please do not prompt the student.
- Please ensure that the station remains tidy and is reset between candidates.

### Further reading

- National Institute for Healthcare Excellence. Head injury: Assessment and early management. NICE Guidelines. 2014 [cited 2022 Jan 18]. Available at: https://www.nice.org.uk/guidance/cg176

### Guidance for examiner

Working definition of competent performance: the candidate effectively completes the task within the allotted time, in a manner that maintains patient safety, even though the execution may not be efficient and well structured.

#### Establishes a good doctor-patient relationship

The *competent candidate* acts within the ethical framework (respects autonomy, justice, non-maleficence, beneficence). In addition, the *good candidate* displays empathy and compassion, acknowledging the patient’s discomfort and the anxiety related to ongoing physical symptoms.

#### Gathering information: history and examination findings

The *competent candidate* gathers sufficient information to identify current medical issues *[mild concussion]* and identify any ongoing biopsychosocial risks. In addition, the *good candidate* explores the patient’s experience, fears *[risk to playing career]* and expectations *[need for ‘brain scan’]*, health-seeking behaviour and identifies opportunities for health promotion *[ongoing risk if playing starts too soon; optimising healthy lifestyle choices].*

#### Clinical judgement

The *competent candidate* interprets the available clinical data (from history, exam and investigation findings) to make the correct working diagnosis *[mild concussion*]. The *good candidate* is able to make a comprehensive three-stage assessment *[as for ‘competent’ + fear of sporting limitations; desire for rapid return to play; appropriate investigations (CT not indicated) and follow-up].*

#### Explaining and planning

The *competent candidate* clearly explains the working diagnosis *(reassurance; no jargon; comprehensive; simple language)* and possible interventions. The *good candidate*, in addition, provides a platform for the patient to engage as an equal partner in sharing information, and decision-making.

#### Management

The *competent candidate* uses current evidence-based guidelines to develop a management plan (*observation in-hospital till GCS 15/15; symptomatic therapy, avoids over-medicating, information-sharing, provides safety netting and clear instructions about red flags, enquires about accessibility to close monitoring at home for next 24hrs, avoid intoxicants until full recovery*). In addition, the *good candidate* develops a comprehensive plan using the biopsychosocial approach (*as for ‘competent’ + counsels the patient on the temporary loss of function and offers assistance with a structured follow-up plan and fit-to-play assessment*).

### Examination findings and investigations

Vitals:

- Blood pressure (BP): 103/68; heart rate (HR): 87/min; respiratory rate (RR): 14/min; body mass index (BMI) 23; temparature: 36.4 °C
- No Jaundice, pallor, lymphadenopathy, clubbing, cyanosis, or oedema

Systemic exam:

- Ear, nose and throat (ENT): no abnormalities of note
- Resp: no abnormalities
- Cardiovascular system (CVS): no abnormalities
- Abd: soft, non-tender
- C-Spine: no observable abnormalities in posture or anatomy; no point tenderness; full and painless active range of motion
- Head examination:
  ■ Early bruising left frontotemporal area – no discernible fracture
  ■ No fluid leakage or bleeding from nose or ears
  ■ No periorbital bruising, bleeding or discolouration
- Central nervous system (CNS):
  ■ Glasgow Coma Scale: (1) Eye movement – 4; (2) Verbal response – 4 (disorientated to place/time); (3) Motor response – 6.
  ■ Cerebellar function: (1) Slightly impaired fine movement control, needs support with balance coordination; (2) No nystagmus; no dysdiadokinesis
  ■ Motor function: power and reflexes normal across all motor systems
  ■ Sensation: all intact
  ■ Cranial nerves, including vision – intact

Blood results:

- Point of care hemoglobin (Hb): 14.7 g/dL
- Point of care random Glucose: 5.6
- Urine dipstick – no abnormalities

### Role play – Instructions for actor

**Appearance and behaviour:** a young man (Siwe) with coach (another adult)

**Opening statement by coach: ‘**Dr, this player had a head injury on the rugby field and was unconscious for about two minutes. I brought him straight here’.

#### History

Siwe is quiet and leaves most of the talking to coach

Open responses: Coach freely tells the doctor:

It was about 25 min into the game, everything was going as per normal, Siwe went in for a tackle and took a knee directly to his head, and then fell to the ground unconscious. They sprinkled his face with cold water and after about 2 min he regained consciousness but was unsteady on his feet, and a bit confused.

He plays at scrumhalf.

This happened about 15 min ago.

You are not aware of any medical problems – he has been playing for you for about 3 years now. His parents are on their way to the hospital.

Closed responses: Only tell the doctor if asked:

- There was no vomiting or fitting.
- He is still a bit unsteady on his feet – you had to help him from the car, although this seems to be better than at the field
- He was quiet in the car, asking what happened and where they were going
- Fears:
- Will this affect his playing? The provincial scout was at the game, as he is being considered for selection to the provincial team
- How long will he have to stay away from the game?
- Expectations:
- Do they need to have a brain scan?

Siwe:

- You are a bit puzzled – why are you here
- Don’t remember how you got here – the last thing you remember is running onto the field to play
- Gives the wrong date and doesn’t know that you are in a hospital EC
- You have a headache, but not nausea
- Your vision is perfectly fine

